# Monitoring Multiple Sexually Transmitted Pathogens Through Wastewater Surveillance

**DOI:** 10.3390/pathogens14060562

**Published:** 2025-06-05

**Authors:** Balghsim Alshehri, Olivia N. Birch, Justin C. Greaves

**Affiliations:** Department of Environmental and Occupational Health, School of Public Health, Indiana University-Bloomington, Bloomington, IN 47408, USA; balsheh@iu.edu (B.A.); onbirch@iu.edu (O.N.B.)

**Keywords:** wastewater-based epidemiology, HIV, STD/STI, chlamydia, gonorrhea, syphilis, hepatitis C virus, herpes

## Abstract

Wastewater-based epidemiology (WBE) offers a promising tool for sexually transmitted infection (STI) surveillance, especially in settings where underdiagnosis or social stigma complicates conventional reporting. To assess its utility, we conducted a year-long study examining six STIs, *Chlamydia trachomatis*, *Treponema pallidum*, *Neisseria gonorrhoeae*, human immunodeficiency virus (HIV), hepatitis C virus (HCV), and herpes simplex virus (HSV), in weekly composite samples from the primary influent of a small-sized Midwestern wastewater treatment plant. Pathogen detection and quantification were performed via digital PCR. Among the tested targets, Gonorrhea, HIV, HCV, and HSV were detected at the highest frequencies, often in 40–50% of the samples, while Chlamydia and Syphilis appeared less frequently. Despite the variability in detection patterns, this study demonstrates that even infrequent signals can reveal community-level shedding of poorly reported or asymptomatic infections. Although month-to-month wastewater data were not strongly correlated with corresponding clinical records, which could potentially reflect delayed healthcare seeking and pathogen-specific shedding dynamics, the overall findings underscore WBE’s ability to complement existing surveillance by capturing infections outside traditional healthcare channels. These results not only advance our understanding of STI prevalence and population shedding but also highlight the practical benefits of WBE as an early warning and targeted intervention tool.

## 1. Introduction

Sexually transmitted infections (STIs) pose a significant public health challenge worldwide, with millions of new cases reported annually [1]. The World Health Organization (WHO) estimates that in 2020, there were approximately 128.5 million new cases of Chlamydia and 7.1 million new cases of Syphilis globally [1]. In the United States, the Centers for Disease Control and Prevention (CDC) recorded around 1.6 million cases of Chlamydia and nearly 250,000 cases of Syphilis in 2022 [2]. If left untreated, these infections can lead to severe complications, including infertility, pelvic inflammatory disease, congenital syphilis, and increased susceptibility to HIV [3]. Despite the availability of effective diagnostic tools and treatments, STIs continue to proliferate due to underdiagnosis, lack of access to testing, and persistent social stigma [4,5].

Traditional STI surveillance methods, such as clinical testing, opportunistic screening, and self-testing, often fail to capture the true prevalence of these infections [6]. Individuals, particularly those in low-income or marginalized communities, may avoid testing due to stigma, misinformation, or limited access to healthcare services [7,8]. The COVID-19 pandemic further exacerbated these challenges by diverting public health resources away from STI programs, leading to reduced testing and delayed diagnoses [9,10,11]. Consequently, there is a pressing need for innovative, non-invasive, and cost-effective approaches to monitoring STIs at the community level [12].

Wastewater-based epidemiology (WBE) has emerged as a powerful tool for monitoring infectious diseases at the population level [13,14]. WBE involves analyzing wastewater samples to detect and quantify biomarkers, including pathogens, pharmaceuticals, and other health-related indicators. This approach offers several advantages over traditional surveillance methods, such as capturing data from asymptomatic individuals, cost-effectiveness, and non-invasiveness [15,16]. WBE has been successfully employed to track a wide range of viral infections, including SARS-CoV-2, influenza, norovirus, and poliovirus [17,18]. More recently, it has also been applied to monitor bacterial pathogens, such as Salmonella and Escherichia coli, providing valuable insights into community disease dynamics [19].

Despite its success in infectious disease surveillance, the application of WBE to STIs remains largely unexplored. A recent study has demonstrated the feasibility of detecting Chlamydia trachomatis and Treponema pallidum (the causative agents of Chlamydia and Syphilis, respectively), in wastewater samples [11]. These findings suggest that WBE could serve as a complementary tool for STI surveillance, aiding in the identification of underreported cases and monitoring disease trends in real-time. In that same study, Zhao et al. (2024) [11] successfully detected and quantified C. trachomatis and T. pallidum in wastewater samples from an urban metro area, revealing potential discrepancies between clinical reports and actual infection prevalence. Similarly, Chin Quee (2023) demonstrated the utility of WBE in detecting C. trachomatis on a university campus [20], highlighting its potential for high-risk populations such as college students.

Given the potential of WBE to address gaps in STI surveillance, this study aims to implement WBE for STI surveillance in the diverse community setting of Bloomington and compare WBE data with clinical surveillance records to understand similarities in data over a one-year study period. Our study specifically advances on previous studies by comprehensively measuring six different STI targets, including Gonorrhea, Chlamydia, Syphilis, human immunodeficiency virus (HIV), hepatitis C virus (HCV), and human simplex virus (HSV), over the course of a year. By advancing WBE methodologies and validating its application for STI monitoring, this research seeks to enhance public health surveillance and inform targeted intervention strategies for STI prevention and control [21].

## 2. Materials and Methodology

### 2.1. Sample Collection and Site Selection

Wastewater samples were collected from the Blucher Poole Wastewater Treatment Plant (WWTP), located in Bloomington, Indiana, which serves a substantial portion of the local population with a daily processing flow exceeding 4.5 million gallons. To ensure comprehensive monitoring, 24-hour composite influent wastewater samples were obtained weekly over a 12-month period, from January to December 2023. Each composite sample comprised 500 mL of untreated influent wastewater, collected in sterile polypropylene bottles and immediately transported in coolers to the laboratory, where they were stored at −20 °C within two hours of collection until processing. 

### 2.2. Sample Concentration and DNA/RNA Extraction

Wastewater samples were processed following a standardized concentration and extraction protocol. Initially, 50 mL aliquots of primary influent wastewater samples were acidified to a pH of 3.5 using hydrochloric acid (HCl) (Fischer Scientific, Hampton, NH, USA). Each sample included a known concentration of bovine coronavirus solution as an extraction efficiency tracer. Samples underwent filtration through mixed cellulose ester membranes (47 mm diameter, 0.45 µm pore size) under vacuum filtration. Post-filtration, membranes were transferred into 2.0 mL PowerBead tubes for immediate or subsequent total nucleic acid (TNA) extraction, utilizing the AllPrep PowerViral DNA/RNA extraction kit (Qiagen, Germantown, MD, USA) following manufacturer guidelines. Eluted total nucleic acids (TNA) were stored at -20 °C pending analysis 

### 2.3. Microbial Quantification by dPCR

Pathogen quantification was conducted using digital PCR (dPCR) with the QIAcuity Four Platform System (Qiagen, Germantown, MD, USA). For RNA targets (HIV and HCV), a one-step reverse transcription digital PCR (RT-dPCR) protocol was used, incorporating Qiacuity OneStep Advanced RT Mix within the QIAcuity RT-PCR alongside 1X QIAcuity Probe PCR mix, 0.8 µM forward and reverse primers, 0.4 µM probe, and 10 µL extracted DNA to enable cDNA synthesis and amplification in a single reaction. Primers and probes specifically for *Chlamydia trachomatis*, *Treponema pallidum*, Gonorrhea, HIV, HCV, and HSV were obtained from Integrated DNA Technologies (IDT, Newark, NJ, USA), incorporating dual-quenched probes (Zen™ internal quencher and 3′ Black Hole™ quencher). While primer and probe sets were selected based on published, peer-reviewed, and validated assays presented in Table S1 in the Supplementary information, formal in silico specificity screening was conducted using NCBI BLAST [22,23,24,25]. Thermal cycling conditions included an initial denaturation at 95 °C for 2 minutes, followed by 45 cycles of denaturation at 95 °C for 5 seconds, and annealing/extension at 60 °C for 30 seconds. Using DNA controls, the lowest detection limit was estimated for all PCR assays to be 1.4 copies/PCR reaction for all targets, which represents 400 GC/L (2.6 log_10_ copies/L) [26,27,28].

### 2.4. Data Analysis

The concentration of viruses (gene copies (gc)/L) was calculated using the following formula: [29]

$$\frac{gc}{L}=\frac{gc}{\mu L}\times \frac{Vol\;PCR\;reaction\;(40\;\mu L)}{Vol.NA\;analyzed\;(10\;\mu L)}\times \frac{Vol.NA\;eluted\;(100\;\mu L)}{vol\;sample\;(0.05\;L)}.$$

Data analyses were conducted using GraphPad Prism v10 software (Boston, MA, USA). The pathogen concentrations were calculated as genome copies per liter (gc/L), applying standard dPCR analytical formulas. Statistical significance between sample groups (e.g., Clinical vs. wastewater concentrations) was assessed through ANOVA tests and Pearson’s correlation, with significance set at *p* < 0.05 [30]. Data visualization included concentration trend graphs and rolling average curves [31]. This methodological approach aimed to ensure a comprehensive, reliable, and reproducible assessment of pathogen dynamics in wastewater, facilitating effective integration of wastewater-based epidemiology into public health surveillance frameworks [32].

### 2.5. Clinical Data

Estimated monthly numbers of incident cases of Chlamydia, Gonorrhea, and HCV at the county level were obtained from publicly available county health department reports for 2023 [33]. Estimates for HIV, Syphilis, and HSV could not be obtained due to case counts falling below the minimum reporting threshold (typically <5 cases/month), which restricts public disclosure to protect privacy. The wastewater concentrations and incident cases for the pathogens we could obtain were compared using Pearson’s R correlation.

## 3. Results and Discussion

### 3.1. Rates of Detection

**Figure 1:**
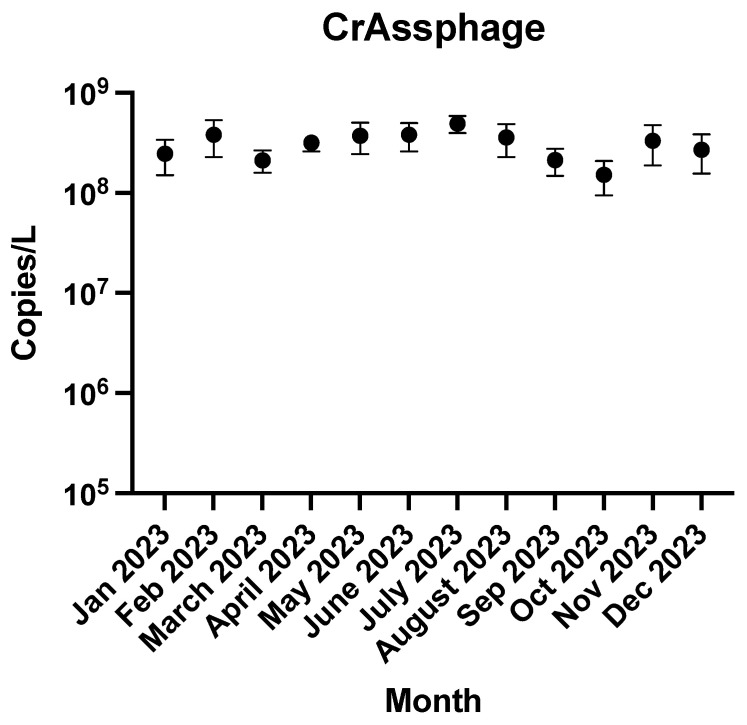
Monthly average concentrations of crAssphage in Bloomington wastewater over a year. The bars are standard deviation for four samples collected each month (n = 4).

Figure 1 shows monthly crAssphage detections and highlights consistently high concentrations throughout the sampling period of 2023. Little variation was recorded between the different months with crAssphage concentrations ranging between 10^8^ and 10^9^ copies/L. There were also no statistically significant differences between the concentrations of each month based on the one-way ANOVA (*p* = 0.88). CrAssphage was included as a fecal strength biomarker to verify consistent sample integrity across timepoints; its relatively stable concentrations suggest minimal variation in fecal loading and validate comparisons of pathogen trends across the sampling period. Table 1 summarizes the detection frequency and mean viral/bacterial concentrations (log_10_ copies/L) for all tested targets (n = 51 samples). Among the six pathogens examined, Gonorrhea and HSV exhibited the highest detection frequency, with 24 (47.1%) positive samples and a mean concentration of 3.08 and 3.00 log_10_ copies/L, respectively. HIV and HCV were each detected in roughly one-half of the tested samples as well (43.1%), and their average concentrations were 2.95 log_10_ copies/L and 3.23 log_10_ copies/L, respectively. By comparison, Chlamydia and Syphilis were each detected in seven (13.7%) and eight samples (15.6%), respectively, with mean concentrations of 2.64 and 2.76 log_10_ copies/L, respectively. 

The higher positivity rates for Gonorrhea, HIV, Hep C, and HSV suggest that these pathogens may be more frequently shed in this community’s wastewater or that their persistence in the sewer network is comparatively greater. Meanwhile, the lower detection frequencies for Chlamydia and Syphilis could reflect multiple factors, such as reduced prevalence, differing shedding patterns, or potentially quicker degradation in the wastewater environment relative to other pathogens. In addition, the comparable log_10_ concentrations observed across each organism point to broadly consistent recovery and measurement efficiencies in the droplet digital PCR assays. 

Although these findings underscore the feasibility of detecting and quantifying a range of sexually transmitted pathogens in wastewater, the divergent positivity rates highlight how WBE might offer insights into both pathogen prevalence and population-level shedding behaviors. Such data may supplement traditional clinical reports, especially for underdiagnosed infections or in instances where case reporting is incomplete. Further investigation into seasonal patterns, the dynamics of pathogen shedding, and quantitative comparisons with clinical surveillance data would help clarify the utility of these wastewater-derived measurements in guiding targeted public health interventions.

### 3.2. Temporal Trends

**Figure 2:**
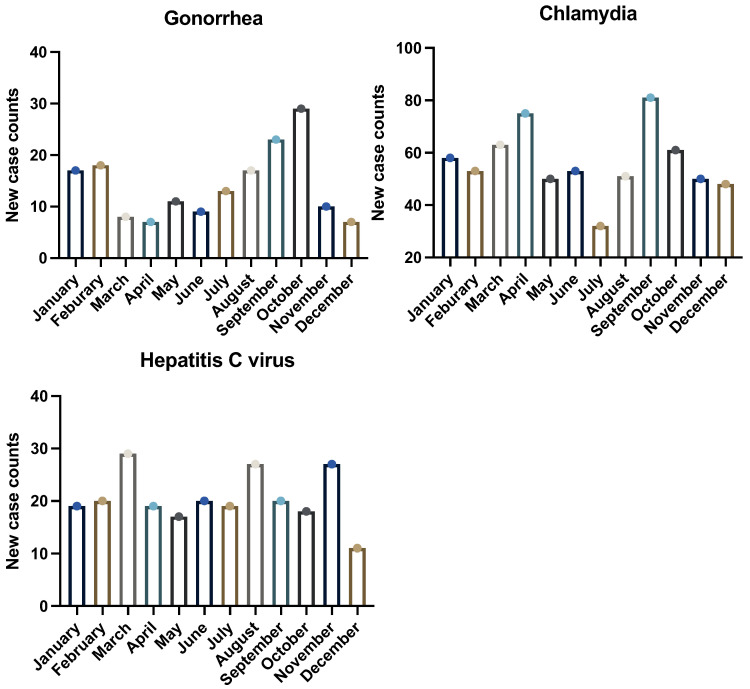
Number of new-case counts by month for Gonorrhea, Chlamydia, and HCV in the county for the year 2023.

**Figure 3:**
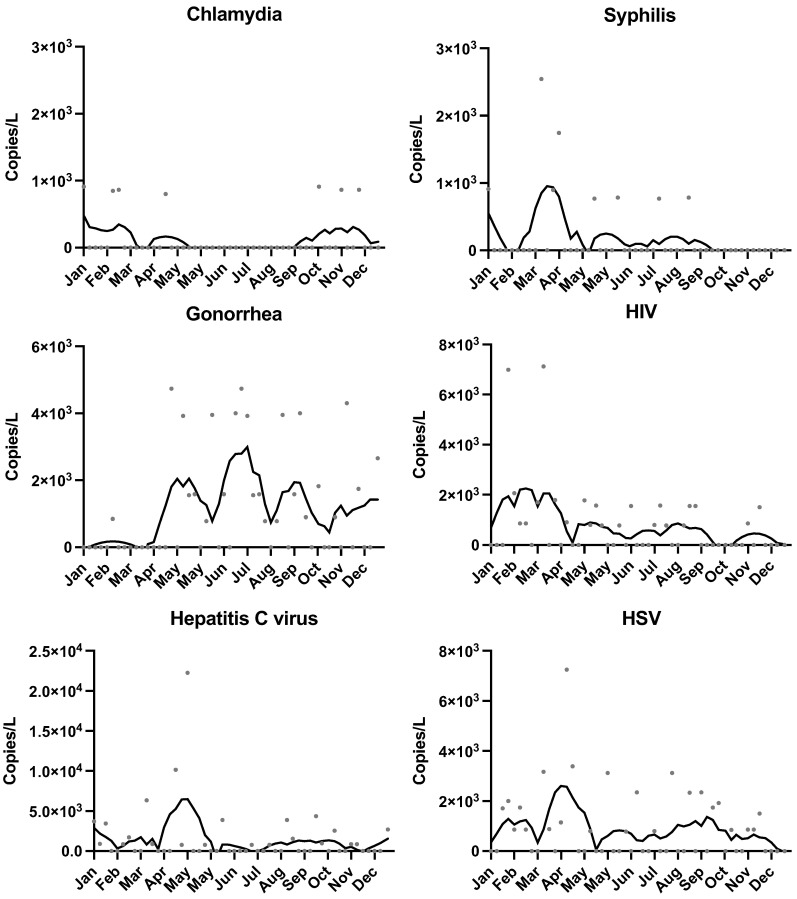
Wastewater concentrations of Chlamydia, Syphilis, Gonorrhea, HIV, Hepatitis C virus, and HSV nucleic acids. The black line represents the 5-sample smoothed and trimmed average.

Clinically, the highest number of Chlamydia cases was reported in September (81 cases), followed by April (75 cases), and the lowest in July (32 cases) (Figure 2). Through WBE, Chlamydia was detected intermittently, with two notable detection periods observed—one in January and one in October—though overall concentrations remained consistent when detected (Figure 3). However, during the remaining months, concentrations were consistently not detected. Syphilis exhibited sporadic detection in wastewater samples, with a high concentration of 3.24 log_10_ copies/L in April and additional detections observed in January and September. No detectable concentrations were observed during the other months. Clinical reports for syphilis indicated minimal recorded cases, consistently fewer than five per month, except for January and April, which reported zero cases. Gonorrhea displayed variable concentrations, with the highest average concentration recorded in September (3.60 log_10_ copies/L), followed by December (3.24 log_10_ copies/L) and August (3.20 log_10_ copies/L). Lower concentrations were noted in June, November, and October. Clinical case reports peaked in October (29 cases) and September (23 cases), with the lowest reported in April and December (7 cases each). 

HIV was detected in wastewater at low but consistent concentrations across most months, with notable increases observed intermittently throughout the year [24]. Peak levels were generally observed in mid-to-late summer, though certain months, such as late spring, also showed transient spikes in HIV genome copies per liter (gc/L). HCV RNA was intermittently but repeatedly detected throughout the monitoring period. Temporal inspection revealed three distinct surges: late February, early July, and mid-October, each spanning two to three consecutive sampling weeks before returning to near or below the detection limit. These episodic peaks were not accompanied by corresponding increases in county-reported incident cases (all months < 5 cases), underscoring the likelihood of silent transmission chains that evade conventional surveillance [34]. HSV (Herpes Simplex Virus) followed a similarly sporadic temporal pattern, with only a few months demonstrating substantial viral loads and other intervals reflecting undetectable or minimal concentrations. These observations highlight potentially complex shedding dynamics for the viral pathogens, in which factors such as active infections, disease management, and treatment adherence could influence wastewater signals [8].

The repeated detection of these viral targets underscores wastewater-based epidemiology’s capability to capture infection signals irrespective of conventional reporting constraints or limited case counts. Additionally, preliminary statistical assessments indicated only modest correlations between HIV and select bacterial STIs (notably syphilis, r = 0.5), but small sample sizes and low clinical reporting rates warrant cautious interpretation. Collectively, these results reinforce the value of incorporating viral pathogen monitoring into WBE for STI surveillance [35]. By capturing fluctuations in community-level infection burdens, particularly for underreported or stigmatized conditions, this approach may offer an early warning system and promote more proactive public health interventions.

Although HCV was detected in roughly half of the samples, its concentrations fluctuated more than any other target, with several weeks falling below the limit of detection, followed by discernible surges. The biology of HCV transmission differs markedly from that of the classical bacterial STIs [36]. Fecal shedding of HCV RNA has been documented in both acute and chronic phases [37], but the magnitude and duration of shedding are highly variable and may be influenced by liver disease stage, antiviral therapy, and gut inflammation [36]. The intermittent detection pattern observed here is therefore biologically plausible and may indicate episodic shedding from a relatively small pool of viremic individuals [38]. These fluctuations likely also result from the combination of other biological factors and possible methodological limitations, including matrix inhibition and the inherent sensitivity threshold of digital PCR.

Similar intermittency has been reported in wastewater studies conducted in regions with established harm-reduction programs, where ongoing transmission occurs in focal networks rather than community-wide outbreaks [39]. 

### 3.3. Comparison with Clinical Data

Using Spearman’s correlation analysis, we tested for correlation between wastewater detections and clinical case counts for Gonorrhea and Chlamydia. Our results showed no significant correlation between clinical case number per month and wastewater detections (r = -0.14 for Chlamydia, R = -0.02 for HCV, and R = -0.05 for Gonorrhea). Clinical data for HIV, Syphilis, and HSV had too few counts for us to compare with wastewater or to obtain from the health department. The absence of sufficient clinical data for these pathogens underscores the need for additional studies with expanded access to de-identified line-list datasets in future investigations.

For the bacterial targets, the correspondence between wastewater signals and county-level case notifications was pathogen-specific. Gonorrhea concentrations rose sharply in late summer and early autumn and coincided with the highest monthly case counts, suggesting that the organism is shed in quantities proportional to community incidence. By contrast, Chlamydia exhibited its highest wastewater loads in mid-winter, several months before the clinical peak, whereas Syphilis appeared only sporadically in wastewater despite persistent low-level clinical activity. These discordances likely reflect a combination of delayed healthcare seeking, differential shedding kinetics, and the physicochemical stability of individual pathogens once excreted [40,41]. Importantly, the absence of statistically significant correlations for Chlamydia and Syphilis does not negate the epidemiological value of WBE; rather, it underscores the need for mechanistic studies that link infection stage, treatment status, and fecal or urogenital shedding rates [42].

The divergence between wastewater and clinical data is unsurprising given the many breakpoints between infection, healthcare seeking, diagnostic confirmation, and public health reporting. Wastewater captures both symptomatic and silent infections, registers them in near-real time, and aggregates the entire catchment [43,44]. Clinical notifications, in contrast, reflect only those who access care, agree to testing, and remain within county lines when results are assigned [11]. Clinical data are often delayed by several weeks due to the time required for healthcare seeking, diagnostic confirmation, and case reporting, which limits real-time alignment with wastewater trends and complicates direct statistical comparisons. 

Delays of two to six weeks between specimen collection and public posting are routine, further blurring synchronicity [45,46]. Additionally, the city’s transient student population adds further complexity, as individuals may receive testing on campus while residing off-campus or outside the wastewater catchment area, introducing spatial mismatch between clinical and environmental data streams. Also, the lack of statistically significant correlations should be interpreted cautiously, as it likely stems from sparse clinical data, inconsistent reporting intervals, and population mobility rather than a true absence of association between wastewater and clinical indicators.

## 4. Conclusions

This investigation is, to our knowledge, the first year-long application of wastewater-based epidemiology (WBE) to a suite of six sexually transmitted pathogens (*Chlamydia trachomatis, Treponema pallidum, Neisseria gonorrhoeae*, HIV, HCV, and HSV) in a small-sized Midwestern city. By utilizing high-resolution dPCR and weekly composite sampling, we were able to capture both the seasonality and stochasticity of pathogen shedding into the municipal sewer network. The findings advance current STI surveillance practice by demonstrating that WBE can recover meaningful signals even for pathogens that are chronically under-reported in routine clinical systems [47].

Several methodological limitations warrant cautious interpretation of our findings. Weekly composite sampling, while logistically feasible, may miss short-lived shedding events, particularly for pathogens such as HCV that exhibit episodic excretion. Future work should integrate richer clinical datasets, including de-identified line-list data for pathogen viral/bacterial load testing, to enable time-series modeling of wastewater and clinical metrics [48]. Future studies should also consider daily or bi-weekly sampling to improve temporal resolution and sensitivity. Incorporating metagenomic sequencing would also allow simultaneous genotyping of various pathogens, providing insight into transmission clusters and antiviral resistance patterns.

Overall, this study demonstrates that WBE can detect and quantify sexually transmitted pathogens, even in settings where routine surveillance yields lower case numbers [49]. The intermittent yet recurrent wastewater signal suggests a persistent reservoir of untreated or recently infected individuals and highlights the added value of environmental monitoring in guiding elimination efforts. By incorporating wastewater data with enhanced clinical reporting and community outreach, public health agencies can move closer to comprehensive, equity-focused control of sexually transmitted and blood-borne infections [29].

## Figures and Tables

**Table 1:** Detection frequency and average (mean ± standard deviation) concentrations for each STI in wastewater over the 12-month study period.

Target Gene	No. of Tested Samples	No. of Positive Samples (%)	Mean Concentration ± Standard Deviation (log_10_ copies/L)
Chlamydia	51	7 (13.7%)	2.64 ± 0.002
Syphilis	51	8 (15.6%)	2.76 ± 0.041
Gonorrhea	51	24 (47.1%)	3.08 ± 0.091
HIV	51	22 (43.1%)	2.95 ± 0.110
Hep C	51	22 (43.1%)	3.23 ± 0.300
HSV	51	24 (47.1%)	3.00 ± 0.089

## Data Availability

All data will be made available upon request.

## Supplementary Materials

The following supporting information can be downloaded at: https://www.mdpi.com/article/10.3390/pathogens14060562/s1, Table S1: Primers and probes for each of the targets used throughout the study [50]

## References

[1] World Health Organization Sexually transmitted infections (STIs) [Internet]. Geneva: World Health Organization; 2025 May 29. https://www.who.int/news-room/fact-sheets/detail/sexually-transmitted-infections-(stis).

[2] Centers for Disease Control and Prevention Sexually Transmitted Infections Surveillance 2022 [Internet]. Atlanta (GA): CDC; 2022. https://www.cdc.gov/sti-statistics/media/pdfs/2024/11/2022-STI-Surveillance-Report-PDF.pdf.

[3] Baraitser P., Alexander S., Sheringham J. (2011). Chlamydia trachomatis screening in young women. Curr. Opin. Obstet. Gynecol..

[4] Lichtenstein B. (2003). Stigma as a barrier to treatment of sexually transmitted infection in the American deep south: Issues of race, gender and poverty. Soc. Sci. Med..

[5] Balfe M., Brugha R., O’Connell E., McGee H., O’Donovan D., Vaughan D. (2010). Why don’t young women go for Chlamydia testing? A qualitative study employing Goffman’s stigma framework. Heal. Risk Soc..

[6] Gitter A., Oghuan J., Godbole A.R., Chavarria C.A., Monserrat C., Hu T., Wang Y., Maresso A.W., Hanson B.M., Mena K.D. (2023). Not a waste: Wastewater surveillance to enhance public health. Front. Chem. Eng..

[7] Cunningham S.D., Kerrigan D.L., Jennings J.M., Ellen J.M. (2009). Relationships Between Perceived STD-Related Stigma, STD-Related Shame and STD Screening Among a Household Sample of Adolescents. Perspect. Sex. Reprod. Health.

[8] Theunissen K.A.T.M., Bos A.E.R., Hoebe C.J.P.A., Kok G., Vluggen S., Crutzen R., Dukers-Muijrers N.H.T.M. (2015). Chlamydia trachomatis testing among young people: What is the role of stigma?. BMC Public Health.

[9] Sentis A., Prats-Uribe A., López-Corbeto E., Montoro-Fernandez M., Nomah D.K., de Olalla P.G., Mercuriali L., Borrell N., Guadalupe-Fernández V., Reyes-Urueña J. (2021). The impact of the COVID-19 pandemic on Sexually Transmitted Infections surveillance data: Incidence drop or artefact?. BMC Public Health.

[10] Philo S.E., Keim E.K., Swanstrom R., Ong A.Q., Burnor E.A., Kossik A.L., Harrison J.C., Demeke B.A., Zhou N.A., Beck N.K. (2021). A comparison of SARS-CoV-2 wastewater concentration methods for environmental surveillance. Sci. Total. Environ..

[11] Zhao L., Guzman H.P., Xagoraraki I. (2024). Tracking Chlamydia and Syphilis in the Detroit Metro Area by Molecular Analysis of Environmental Samples. Environ. Sci. Technol..

[12] Hart O.E., Halden R.U. (2020). Computational analysis of SARS-CoV-2/COVID-19 surveillance by wastewater-based epidemiology locally and globally: Feasibility, economy, opportunities and challenges. Sci. Total Environ..

[13] Medema G., Heijnen L., Elsinga G., Italiaander R., Brouwer A. (2020). Presence of SARS-Coronavirus-2 RNA in Sewage and Correlation with Reported COVID-19 Prevalence in the Early Stage of the Epidemic in The Netherlands. Environ. Sci. Technol. Lett..

[14] Ahmed W., Angel N., Edson J., Bibby K., Bivins A., O’Brien J.W., Choi P.M., Kitajima M., Simpson S.L., Li J. (2020). First confirmed detection of SARS-CoV-2 in untreated wastewater in Australia: A proof of concept for the wastewater surveillance of COVID-19 in the community. Sci. Total Environ..

[15] Choi P.M., Tscharke B.J., Donner E., O’Brien J.W., Grant S.C., Kaserzon S.L., Mackie R., O’Malley E., Crosbie N.D., Thomas K.V. (2018). Wastewater-based epidemiology biomarkers: Past, present and future. TrAC Trends Anal. Chem..

[16] Mao K., Zhang H., Pan Y., Yang Z. (2021). Biosensors for wastewater-based epidemiology for monitoring public health. Water Res..

[17] Peccia J., Zulli A., Brackney D.E., Grubaugh N.D., Kaplan E.H., Casanovas-Massana A., Ko A.I., Malik A.A., Wang D., Wang M. (2020). Measurement of SARS-CoV-2 RNA in wastewater tracks community infection dynamics. Nat. Biotechnol..

[18] Hovi T., Shulman L.M., VAN DER Avoort H., Deshpande J., Roivainen M., DE Gourville E.M. (2011). Role of environmental poliovirus surveillance in global polio eradication and beyond. Epidemiology Infect..

[19] Hendriksen R.S., Munk P., Njage P., Van Bunnik B., McNally L., Lukjancenko O., Röder T., Nieuwenhuijse D., Pedersen S.K., Kjeldgaard J. (2019). Global monitoring of antimicrobial resistance based on metagenomics analyses of urban sewage. Nat. Commun..

[20] Chin Quee J.E. (2023). Using Wastewater-Based Epidemiology to Study Chlamydia Occurrence on a College Campus.

[21] Gonzalez R., Curtis K., Bivins A., Bibby K., Weir M.H., Yetka K., Thompson H., Keeling D., Mitchell J., Gonzalez D. (2020). COVID-19 surveillance in Southeastern Virginia using wastewater-based epidemiology. Water Res..

[22] Wu Z., Greaves J., Arp L., Stone D., Bibby K. (2020). Comparative fate of CrAssphage with culturable and molecular fecal pollution indicators during activated sludge wastewater treatment. Environ. Int..

[23] Chia C.T., Bender A.T., Lillis L., Sullivan B.P., Martin C.D., Burke W., Landis C., Boyle D.S., Posner J.D. (2022). Rapid detection of hepatitis C virus using recombinase polymerase amplification. PLoS ONE.

[24] Wolfe M.K., Varkila M.R.J., Zulli A., Parsonnet J., Boehm A.B. (2024). Detection and quantification of human immunodeficiency virus-1 (HIV-1) total nucleic acids in wastewater settled solids from two California communities. Appl. Environ. Microbiol..

[25] Aitlhaj-Mhand R., Qasmaoui A., Bellaji B., Remz C., Charof R., El Jaoudi R., Abdelmoumen H., Hançali A., Oumzil H. (2024). Promoting molecular diagnostic equity: Assessing in-house real-time PCR for Neisseria gonorrhoeae in anal samples from MSM recruited in an outpatient setting in Morocco. Infez. Med..

[26] Feng S., Roguet A., McClary-Gutierrez J.S., Newton R.J., Kloczko N., Meiman J.G., McLellan S.L. (2021). Evaluation of Sampling, Analysis, and Normalization Methods for SARS-CoV-2 Concentrations in Wastewater to Assess COVID-19 Burdens in Wisconsin Communities. ACS ES&T Water.

[27] McCall C., Wu H., O’brien E., Xagoraraki I. (2021). Assessment of enteric viruses during a hepatitis outbreak in Detroit MI using wastewater surveillance and metagenomic analysis. J. Appl. Microbiol..

[28] Wilhelm A., Schoth J., Meinert-Berning C., Bastian D., Blum H., Elsinga G., Graf A., Heijnen L., Ho J., Kluge M. (2023). Interlaboratory comparison using inactivated SARS-CoV-2 variants as a feasible tool for quality control in COVID-19 wastewater monitoring. Sci. Total. Environ..

[29] Anderson-Coughlin B.L., Craighead S., Kelly A., Gartley S., Vanore A., Johnson G., Jiang C., Haymaker J., White C., Foust D. (2021). Enteric Viruses and Pepper Mild Mottle Virus Show Significant Correlation in Select Mid-Atlantic Agricultural Waters. Appl. Environ. Microbiol..

[30] Azzellino A., Pellegrinelli L., Pedrini R., Turolla A., Bertasi B., Binda S., Castiglioni S., Cocuzza C.E., Ferrari F., Franzetti A. (2025). Evaluating Interlaboratory Variability in Wastewater-Based COVID-19 Surveillance. Microorganisms.

[31] He Z., Dunne D. (2022). Refining the scope of Journal of Hazardous Materials. J. Hazard. Mater..

[32] Banadaki M.D., Torabi S., Rockward A., Strike W.D., Noble A., Keck J.W., Berry S.M. (2024). Simple SARS-CoV-2 concentration methods for wastewater surveillance in low resource settings. Sci. Total. Environ..

[33] Health, I.D.o. STI Morbidity Dashboard. 2025. https://www.in.gov/health/hiv-std-viral-hepatitis/sexually-transmitted-disease-prevention-program/stds-dashboard/.

[34] Hillary L.S., Farkas K., Maher K.H., Lucaci A., Thorpe J., Distaso M.A., Gaze W.H., Paterson S., Burke T., Connor T.R. (2021). Monitoring SARS-CoV-2 in municipal wastewater to evaluate the success of lockdown measures for controlling COVID-19 in the UK. Water Res..

[35] Nam J.-Y., Jwa E., Eom H., Kim H., Hwang K., Jeong N. (2021). Enhanced energy recovery using a cascaded reverse electrodialysis stack for salinity gradient power generation. Water Res..

[36] Manns M.P., But M., Gane E., Pawlotsky J.-M., Razavi H., Terrault N., Younossi Z. (2017). Hepatitis C virus infection. Nat. Rev. Dis. Primers.

[37] Beld M., Sentjens R., Rebers S., Weel J., Dillen P.W.-V., Sol C., Boom R. (2000). Detection and Quantitation of Hepatitis C Virus RNA in Feces of Chronically Infected Individuals. J. Clin. Microbiol..

[38] Reyne M.I., Allen D.M., Levickas A., Allingham P., Lock J., Fitzgerald A., McSparron C., Nejad B.F., McKinley J., Lee A. (2022). Detection of human adenovirus F41 in wastewater and its relationship to clinical cases of acute hepatitis of unknown aetiology. Sci. Total. Environ..

[39] Guo Y., Li J., O’Brien J., Sivakumar M., Jiang G. (2022). Back-estimation of norovirus infections through wastewater-based epidemiology: A systematic review and parameter sensitivity. Water Res..

[40] Meyer-Weitz A., Reddy P., Borne H.V.D., Kok G., Pietersen J. (2000). Health care seeking behaviour of patients with sexually transmitted diseases: Determinants of delay behaviour. Patient Educ. Couns..

[41] Rotchford K., Strum A.W., Wilkinson D. (2000). Effect of coinfection with STDs and of STD treatment on HIV shedding in genital-tract secretions: Systematic review and data synthesis. Sex. Transm. Dis..

[42] Betancourt W.Q., Schmitz B.W., Innes G.K., Prasek S.M., Brown K.M.P., Stark E.R., Foster A.R., Sprissler R.S., Harris D.T., Sherchan S.P. (2021). COVID-19 containment on a college campus via wastewater-based epidemiology, targeted clinical testing and an intervention. Sci. Total. Environ..

[43] Kilaru P., Hill D., Anderson K., Collins M.B., Green H., Kmush B.L., Larsen D.A. (2022). Wastewater Surveillance for Infectious Disease: A Systematic Review. Am. J. Epidemiology.

[44] Bivins A., Kaya D., Ahmed W., Brown J., Butler C., Greaves J., Leal R., Maas K., Rao G., Sherchan S. (2022). Passive sampling to scale wastewater surveillance of infectious disease: Lessons learned from COVID-19. Sci. Total. Environ..

[45] Hook E.W., Richey C.M., Leone P., Bolan G., Spalding C., Henry K., Clarke P., Smith M., Celum C.L. (1997). Delayed presentation to clinics for sexually transmitted diseases by symptomatic patients—A potential contributor to continuing STD morbidity. Sex. Transm. Dis..

[46] Kersh E.N., Shukla M., Raphael B.H., Habel M., Park I. (2021). At-Home Specimen Self-Collection and Self-Testing for Sexually Transmitted Infection Screening Demand Accelerated by the COVID-19 Pandemic: A Review of Laboratory Implementation Issues. J. Clin. Microbiol..

[47] Farkas K., Cooper D.M., McDonald J.E., Malham S.K., de Rougemont A., Jones D.L. (2018). Seasonal and spatial dynamics of enteric viruses in wastewater and in riverine and estuarine receiving waters. Sci. Total Environ..

[48] Huisman J.S., Scire J., Caduff L., Fernandez-Cassi X., Ganesanandamoorthy P., Kull A., Scheidegger A., Stachler E., Boehm A.B., Hughes B. (2022). Wastewater-Based Estimation of the Effective Reproductive Number of SARS-CoV-2. Environ. Health Perspect..

[49] Karthikeyan S., Ronquillo N., Belda-Ferre P., Alvarado D., Javidi T., Longhurst C.A., Knight R. (2021). High-Throughput Wastewater SARS-CoV-2 Detection Enables Forecasting of Community Infection Dynamics in San Diego County. mSystems.

[50] Brisebois E., Veillette M., Dion-Dupont V., Lavoie J., Corbeil J., Culley A., Duchaine C. (2018). Human viral pathogens are pervasive in wastewater treatment center aerosols. J. Environ. Sci. (China)..

