# Supplementary material for: Monitoring Multiple Sexually Transmitted Pathogens Through Wastewater Surveillance
Source: Pathogens. 2025 Jun 5;14(6):562. doi: 10.3390/pathogens14060562 (PMC12195674; doi:10.3390/pathogens14060562)
Supplement: Supplementary file 1 [file pathogens-14-00562-s001.zip › pathogens-3642950-supplementary.pdf]

Supporting Information: **Monitoring Multiple Sexually Transmitted Pathogens Through Wastewater Surveillance**

Balghsim Alshehri[a], Olivia N. Birch[a], and Justin C.J. Greaves[a]

[a]Department of Environmental and Occupational Health, School of Public Health Indiana University-Bloomington

Corresponding Author:

Justin C.J. Greaves

jcgreave@iu.edu

Indiana University-School of Public Health

2719 E 10th Street, Innovation Center

Bloomington Indiana, 47408

Keywords

Wastewater-based epidemiology; HIV; STD/STI; chlamydia; gonorrhea; syphilis; hepatitis C virus; herpes

Methods:

Table S1: Primers and probes for each of the targets used throughout the study.

Target	Type	Sequence	Source
HIV	Forward	GGCTAACTAGGGAACCCACTG	[24]
	Reverse	TCCACACTGACTAAAAGGGTCTGA	
	Probe	CACTCAAGGCAAGCTTTATTGAGGC	
Hepatitis C virus	Forward	GCAGAAAGCGTCTAGCCATGGCGT	[23]
	Reverse	CTCGCAAGCACCCTATCAGGCAGT	
	Probe	CATAGTGGTCTGCGGAACCGGTGAGT	
C. trachomatis	Forward	CATGARTGGCAAGCAAGTTTA	[11]
	Reverse	GCAATACCGCAAGATTTTCTAG	
	Probe	TGTTCACTCCYTACATTGGAGT	
T. pallidum	Forward	GGTAGAAGGGAGGGCTAGTA	[11]
	Reverse	CTAAGATCTCTATTTTCTATAGGTATGG	
	Probe	ACACAGCACTCGTCTTCAACTCC	
Gonorrhea	Forward	CCGGAACTGGTTTCATCTGATT	[25]
	Reverse	GTTTCAGCGGCAGCATTCA	
	Probe	CGTGAAAGTAGCAGGCGTATAGGCGGACTT	
HSV	Forward	CGCATCAAGACCACCTCCTC	[50]
	Reverse	GCTCGCACCACGCGA	
	Probe	TGGCAACGCGGCCCAAC	
CrAssphage	Forward	CAG AAG TAC AAA CTC CTA AAA AAC GTA GAG	[22]
	Reverse	GAT GAC CAA TAA ACA AGC CAT TAG C	
	Probe	AAT AAC GAT TTA CGT GAT GTA AC	

Citations

24. Wolfe, M.K., et al., *Detection and quantification of human immunodeficiency virus-1 (HIV-1) total nucleic acids in wastewater settled solids from two California communities.* Appl Environ Microbiol, 2024. **90**(12): p. e0147724.

23. Chia, C.T., et al., *Rapid detection of hepatitis C virus using recombinase polymerase amplification.* PLoS One, 2022. **17**(10): p. e0276582.

11. Zhao, L., H.P. Guzman, and I. Xagoraraki, *Tracking Chlamydia and Syphilis in the Detroit Metro Area by Molecular Analysis of Environmental Samples.* Environ Sci Technol, 2024. **58**(40): p. 17606-17616.

25. Aitlhaj-Mhand, R., et al., *Promoting molecular diagnostic equity: assessing in-house real-time PCR for Neisseria gonorrhoeae in anal samples from MSM recruited in an outpatient setting in Morocco.* Infez Med, 2024. **32**(3): p. 352-362.

50. Brisebois, E., et al., *Human viral pathogens are pervasive in wastewater treatment center aerosols.* J Environ Sci (China), 2018. **67**: p. 45-53.

22. Wu, Z.Y., et al., *Comparative fate of CrAssphage with culturable and molecular fecal pollution indicators during activated sludge wastewater treatment.* Environment International, 2020. **136**.
